# Survival of Hepatitis C Virus in Syringes Is Dependent on the Design of the Syringe-Needle and Dead Space Volume

**DOI:** 10.1371/journal.pone.0139737

**Published:** 2015-11-04

**Authors:** Mawuena Binka, Elijah Paintsil, Amisha Patel, Brett D. Lindenbach, Robert Heimer

**Affiliations:** 1 Department of Epidemiology of Microbial Diseases, Yale School of Public Health, New Haven, Connecticut, United States of America; 2 Departments of Pediatrics & Pharmacology, Yale School of Medicine, New Haven, Connecticut, United States of America; 3 Department of Microbial Pathogenesis, Yale School of Medicine, New Haven, Connecticut, United States of America; 4 Center for Interdisciplinary Research on AIDS, Yale University, New Haven, Connecticut, United States of America; UMR Inserm U1052 / CNRS 5286, FRANCE

## Abstract

**Background:**

Many people who inject drugs (PWID) use syringes with detachable needles, which have high dead space (HDS). Contaminated HDS blood may substantially contribute to the transmission of HIV, hepatitis C (HCV), and other blood-borne viruses within this population. Newly designed low dead space (LDS) syringe-needle combinations seek to reduce blood-borne virus transmission among PWID. We evaluated the infectivity of HCV-contaminated residual volumes recovered from two LDS syringe-needle combinations.

**Methods:**

We tested two different design approaches to reducing the dead space. One added a piston to the plunger; the other reduced the dead space within the needle. The two approaches cannot be combined. Recovery of genotype-2a reporter HCV from LDS syringe-needle combinations was compared to recovery from insulin syringes with fixed needles and standard HDS syringe-needle combinations. Recovery of HCV from syringes was determined immediately following their contamination with HCV-spiked plasma, after storage at 22°C for up to 1 week, or after rinsing with water.

**Results:**

Insulin syringes with fixed needles had the lowest proportion of HCV-positive syringes before and after storage. HCV recovery after immediate use ranged from 47%±4% HCV-positive 1 mL insulin syringes with 27-gauge ½ inch needles to 98%±1% HCV-positive HDS 2 mL syringes with 23-gauge 1¼ inch detachable needles. LDS combinations yielded recoveries ranging from 65%±5% to 93%±3%. Recovery was lower in combinations containing LDS needles than LDS syringes. After 3 days of storage, as much as 6-fold differences in virus recovery was observed, with HCV recovery being lower in combinations containing LDS needles. Most combinations with detachable needles required multiple rinses to reduce HCV infectivity to undetectable levels whereas a single rinse of insulin syringes was sufficient.

**Conclusions:**

Our study, the first to assess the infectivity of HCV in residual volumes of LDS syringes and needles available to PWID, demonstrates that LDS syringe-needle combination still has the greater potential for HCV transmission than insulin syringes with fixed needles. Improved LDS designs may be able to further reduce HCV recovery, but based on the designed tested, LDS needles and syringes remain intermediate between fixed-needle syringes and HDS combinations in reducing exposure to HCV.

## Introduction

There are between 11 and 21 million people who inject drugs (PWID) worldwide [1, 2]. PWIDs are at high risk for infection with blood borne pathogens such as hepatitis C virus (HCV) and human immunodeficiency virus (HIV) [3–5]. HCV prevalence rates in PWID range from 40% to 90% [1, 2, 6]. HCV transmission within the PWID population is attributed to the sharing of syringes and other injection paraphernalia [2, 5, 7–11]. In some locations, for the injection of some types and formulations of drugs, PWID prefer syringes with larger volumes and detachable needles [11–13]. These retain larger amounts of residual liquid than low volume syringes with fixed needles, a property that is recognized as a potential contributing factor to the high rates of HCV transmission within this population [2, 10–14].

The volume of the residual fluid within the syringes is dependent on several factors, including needle size and length, the amount of space remaining in the hub of the syringe once the needle is attached, and whether or not the needles are detachable from the syringe barrel [10, 11, 14]. Syringes with fixed needles generally have a ≤1 mL fluid capacity that retain ≤5 μl of liquid after the plunger is fully depressed; high dead space (HDS) syringes with detachable needles, on the other hand, come in volumes of 1 mL or larger and retain more liquid after use [10, 11, 14]. HDS syringes provide a number of advantages for drug preparation and injection. Larger syringe volumes are necessary for the injection of certain drugs, including homemade drug mixtures, viscous liquids such as steroids, and dissolved pharmaceuticals [11, 12]. Larger needle sizes and lengths available for HDS syringes allows for access to deeper veins or intramuscular injection [11]. Finally, detachable needles allow for the filtration of drug mixtures and the possibility of needle replacement should clogging or dulling occur [11]. Given the many functional advantages that HDS syringes have over LDS syringes, it is not surprising that PWID in some circumstances have a preference for HDS syringes and are fairly resistant to making the switch to syringes with fixed needles [11–13].

**Fig 1:**
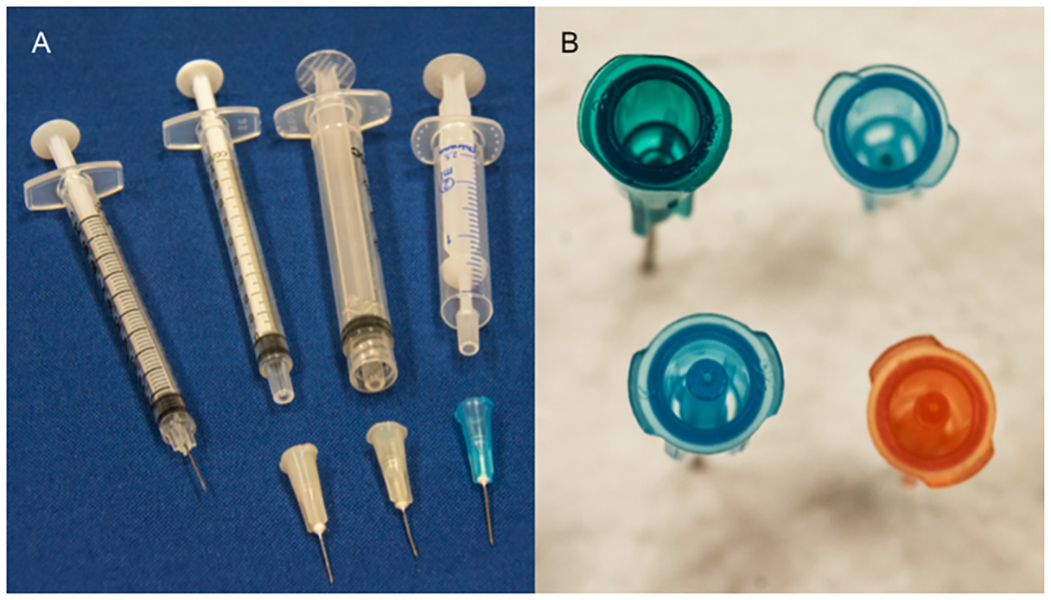
Comparison of Syringe Combinations Tested in This Study. (A) From left to right, the photo shows an insulin syringe with attached needle, a 1 ml tuberculin syringe with detachable needle, a 3 ml Luer-lock syringe with detachable needle, a 2.5 Noloss syringe with the piston withdrawn from the hub of the syringe. (B) Going clockwise from the upper left, the photo shows a 23 gauge, 1¼” standard needle, a 25 gauge, 5/8” standard needle, a 25 gauge, 5/8” lower dead space (LDS) needle, and a 23 gauge, 1¼” standard lower dead space (LDS) needle. Note the addition plastic inside the LDS needles that serve to reduce the dead space. Photographs courtesy of Michael Greenwood.

There is a recent push by harm reduction agencies, syringe manufacturers and distributors to develop alternatives to HDS syringes that fulfill the various needs of PWID [11]. Designs that seek to create low deep space (LDS) syringe and detachable needle combinations have been manufactured [11]. For instance, the Total Dose™ LDS needles, which attach to 2 mL Nevershare^®^ standard syringes, are now available through Exchange Supplies in the United Kingdom [15]. The 2 mL Noloss LDS syringes, in which a piston is attached to the plunger of the syringe that extends through to the tip of the syringe barrel, are available at Apothicom, a French harm reduction supply company [16]. These syringe/needle combinations are shown (Fig 1).

Encouraging PWID to use these LDS syringes and needles requires the demonstration that their use could result in relatively less exposure to HCV in comparison with standard HDS syringes and needles. In this study, we use our previously established microculture assay to determine HCV stability in Total Dose™ LDS needles, used in conjunction with 2 mL Nevershare^®^ standard syringes, and in 2 mL Noloss LDS syringes attached to standard PrecisionGlide™ needles. Comparison was made to standard HDS combinations and to fixed needle syringes intended for insulin injection.

## Materials and Methods

### Virus and Cells

The Jc1/GLuc2A genotype 2a reporter virus, with an inserted luciferase gene from *Gaussia princeps*, was used in these assays [17–19]. Viral stocks and virus titers were obtained as reported previously [20, 21]. Huh-7.5 human hepatoma cells [22] were maintained at 37°C in Dulbecco’s Modified Eagle’s Medium (Invitrogen, Life Technologies, Grand Island, NY) supplemented with 1 mM non-essential amino acids (Invitrogen, Life Technologies, Grand Island, NY) and 10% heat-inactivated fetal calf serum (Omega Scientific, Tarzana, CA) and in the presence of 5% carbon dioxide [20, 21].

### Syringes and Needles

We tested the following syringes: the 2 mL Noloss LDS syringes (Apothicom, Paris, France), the 2 mL Nevershare^®^ syringes (Exchange Supplies, Dorchester, United Kingdom), the 1 mL standard tuberculin syringes (Terumo Medical, Somerset, NJ) and the 1 mL U-100 insulin syringes with fixed 27-gauge ½ inch needles (Terumo Medical, NJ).

We tested PrecisionGlide™ standard needles (Becton, Dickson and Company, Franklin Lakes, NJ), which attached to standard tuberculin and Noloss LDS syringes, and Total Dose™ LDS needles (Exchange Supplies, United Kingdom), which attach only to the Nevershare^®^ syringes. The PrecisionGlide™ standard needle sizes tested were 27-gauge ½ inch (27G½”), 25-gauge ⅝ inch (25G⅝”) and 23-gauge 1¼ inch (23G1¼”) while the Total Dose™ LDS needle sizes were 25G⅝” and 23G1¼”. The higher the gauge, the smaller the inner diameter of the needle bore [23], therefore, the order of bore diameters was, from smallest to largest: 27G, 25G, and 23G.

### Residual liquid in syringes-needle combinations

We estimated the residual liquid in the hub of the different syringe-needle combinations using a 0.003 g/mL solution of brilliant yellow dye (Sigma-Aldrich, St. Louis, MO). Briefly, the dye solution was introduced into different syringe-needle pairs, the plunger was fully depressed, and the syringes were rinsed with 1 mL of distilled water, which was transferred into quartz cuvettes (World Precision Instruments, Sarasota, FL). Absorbance measurements were made at 260nm using a spectrophotometer (GeneQuant™ Pro, Amersham Biosciences, GE Healthcare, Piscataway, NJ). The relative volumes of residual liquid in the syringe-needle combinations were calculated from the respective concentrations of brilliant yellow in the 1 ml rinse water by comparison to a standard curve based on 1:2 serial dilutions of the dye solution. Ten syringes were tested for each syringe-needle pair and data is shown as residual volume ±SD.

### Viability of HCV in syringes

**Fig 2:**
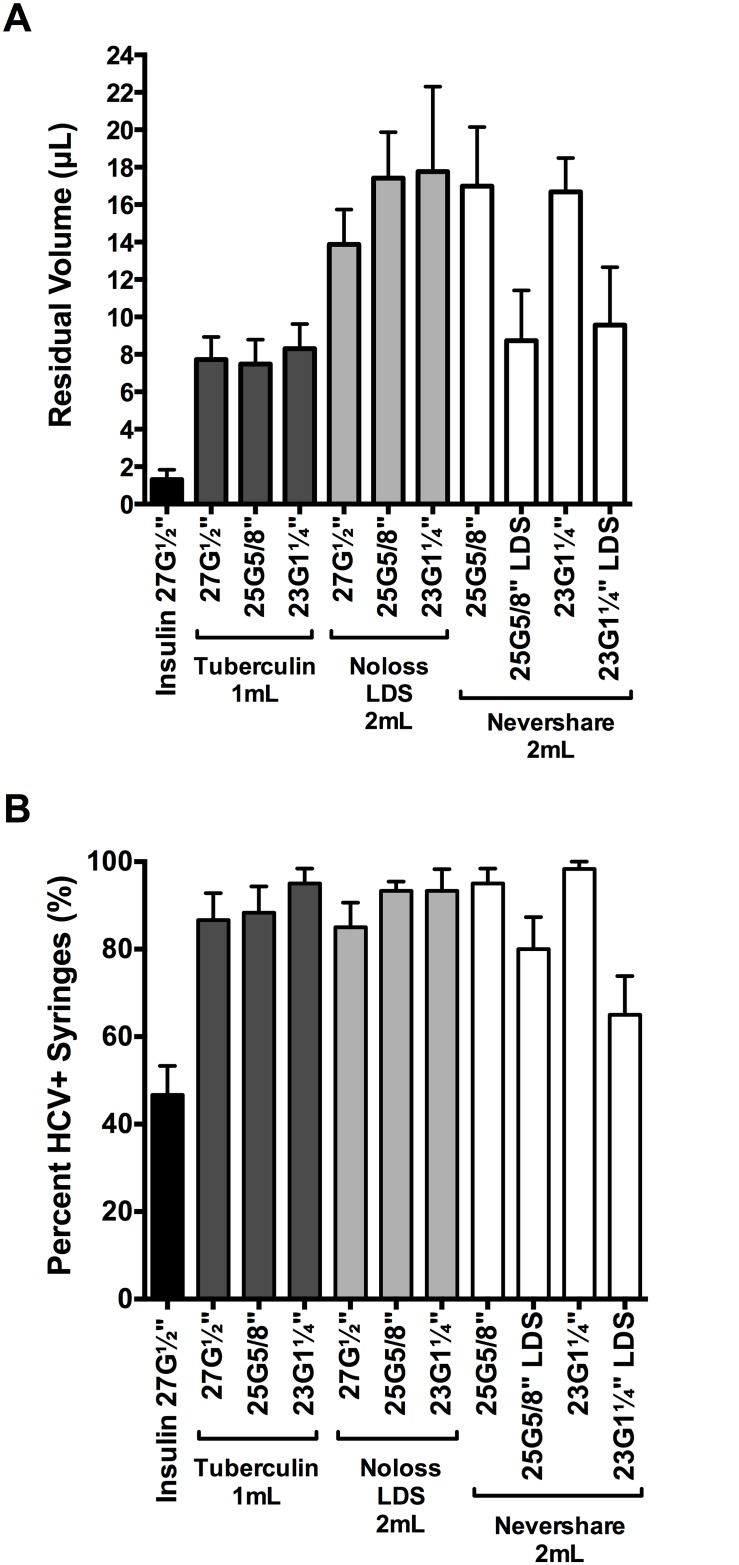
Flow diagram of syringe testing. Syringe-needle pairs were contaminated with virus and tested for viable HCV immediately after contamination, after storage at room temperature or after rinsing with water.

The flow diagram (Fig 2) outlines how experiments were conducted. We tested for HCV infectivity in the syringe-needle combinations immediately after contaminating them with HCV and after storage at room temperature for up to 1 week as described in previous studies [20, 21, 24]. Syringes were loaded with plasma spiked with HCV and stored for up to 7 days at room temperature. The syringe-needle combinations were then flushed with 200 μL of cell culture media, which was then used to infect Huh-7.5 cells seeded at 1.5x10^4^ cells per well in 96-well plates on the previous day. After 5 hours of incubation at 37°C, the cells were washed once with 100 μL sterile phosphate-buffered saline (PBS, Invitrogen, Life Technologies, Grand Island, NY) and 100 μL of fresh cell culture media was added. Viral supernatant was harvested after incubation for 3 days at 37°C and residual infectivity was measured as a function of the amount of luciferase produced in a luciferase-based reporter assay (Promega, Madison, WI). Luminescence measurements were made with a luminometer (Synergy HT, BioTek, Winooski, VT). Outputs from the luminometer are reported as relative luciferase units (RLUs), which quantify the amount of light generated by the virally-encoded luciferase. Previous work has established that the relationship between RLU and HCV infectivity are linear over a range of at least four orders of magnitude of HCV concentration [20]. Half-life data from the storage experiments were obtained by fitting one-phase exponential decay curves to the percent-positive syringe data using GraphPad Prism 6.0 (GraphPad Software, San Diego, CA).

In addition, we tested for HCV infectivity after rinsing contaminated syringes with water. The syringe-needle combinations were loaded with plasma spiked with HCV and then rinsed once or twice with sterile distilled water. The syringes were rinsed with water loaded up to approximately half the volume marked on the syringe barrel, i.e., 2 mL syringes were rinsed with 1 mL of water and 1 mL syringes were rinsed with 0.5 mL of water. The syringe-needle combinations were then flushed with 200 μL of cell culture medium that was added to Huh 7.5 cells. Cultures were maintained and viral infectivity was determined after 3 days as described above.

These experiments were repeated at least three times with a set of 10 syringes tested per condition for each experiment. Residual infectivity was determined as the infectivity above the pre-determined cutoff of 1000 RLU; twice the average background RLU measurements [21, 24]. The results are reported first as the number and percentage of HCV-positive syringes. For those syringes with residual infectivity above the cut-off, the mean residual infectivity (RLU) was calculated across experiments and the data presented with 95% confidence intervals.

### Statistical Analyses

Comparisons of residual volumes between different syringe combinations were made using t-tests. Pairwise comparisons of the proportion of syringes yielding viable HCV in LDS and HDS syringe-needle combinations were made using chi-square tests. Comparisons of residual infectivity in LDS syringe-needle combinations to their HDS counterpart were made using t-tests. Statistical calculations were done with GraphPad Prism 6.0.

## Results

### Relative amounts of residual liquid in different syringe-needle combinations

**Fig 3:**
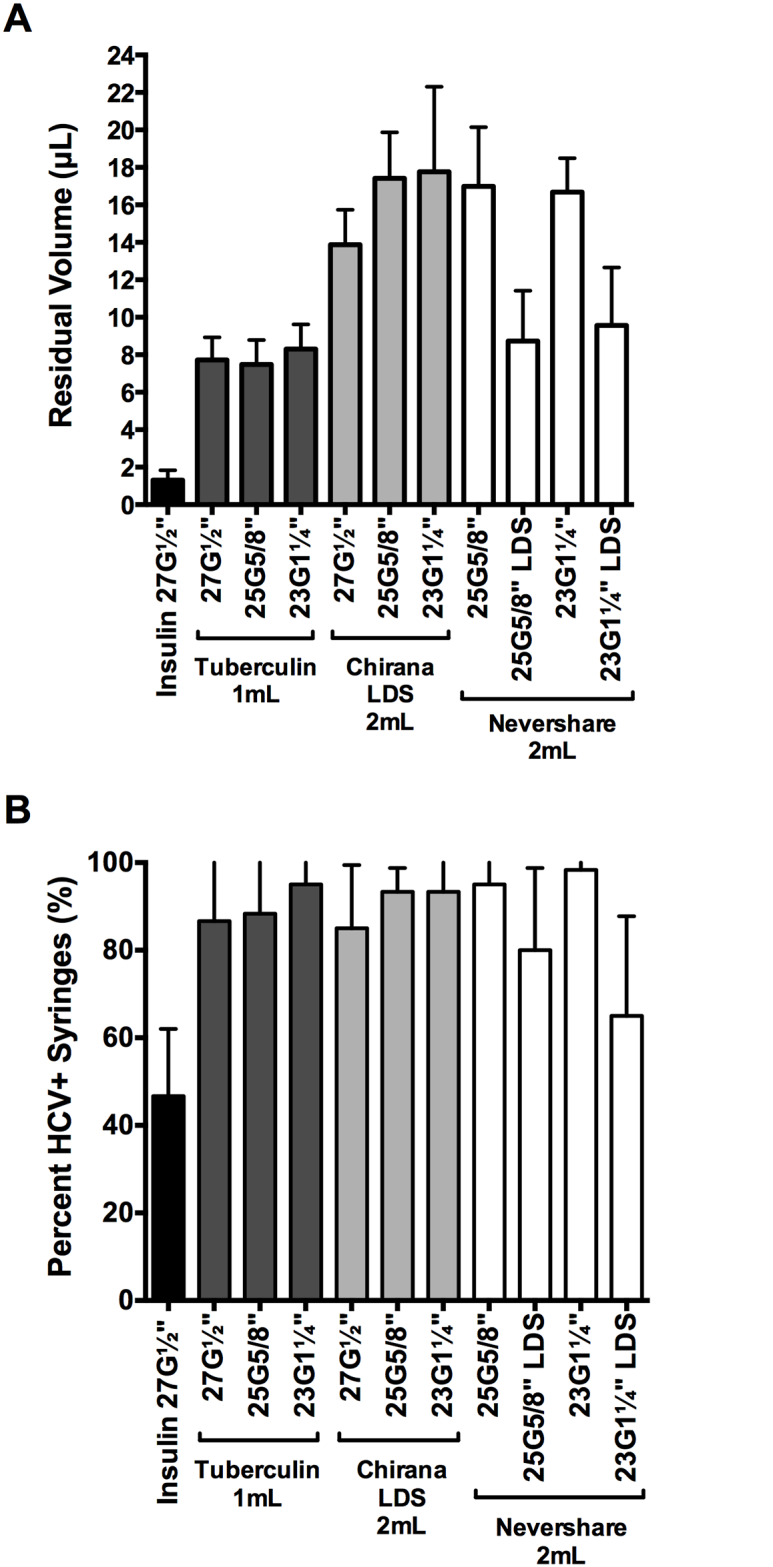
Residual liquid and residual viable HCV in LDS and HDS syringe-needle combinations. (A) Syringe-needle pairs were rinsed with brilliant yellow dye solution and residual volumes were determined with absorbance measurements at 260nm. Syringe-needle pairs were also loaded with plasma spiked with HCV and (B) the frequency of HCV-positive syringes was determined. Each data point denotes the average residual volumes ±SD from 3 syringes or the percentage of HCV-positive syringes ±95% C.I. from at least 3 experiments. G = gauge. LDS = low dead space.

We assessed the relative amounts of residual liquid in the different syringe-needle combinations using the brilliant yellow dye (Fig 3A). Insulin syringes with fixed 27G½” needles retained significantly lower amounts of dye than every syringe with detachable needle tested (1.3 μL ±0.5 μL; 2-tailed t-tests, p<0.0001; Fig 3A). Residual volumes retained in the different 1 mL and 2 mL syringes with detachable needles ranged from 7.5 μL ±1.5 μL (1 mL tuberculin+25G⅝” standard needle) to 17.8 μL ±4.5 μL (2 mL Noloss LDS+23G1¼” standard needle; Fig 3A). The 2 mL standard Nevershare^®^ syringes with detachable standard needles (25G⅝”: 17.0 μL ±3.2 μL, 23G1¼”: 16.7 μL ±1.8 μL residual volume) and the 2 mL Noloss LDS syringes with detachable standard needles (27G½”: 13.9 μL ±1.9 μL, 25G⅝”: 17.4 μL ±2.5 μL, 23G1¼”: 17.8 μL ±4.5 μL residual volume) had significantly higher residual volumes than 1 mL tuberculin syringes with identical needles (27G½”: 7.7 μL ±1.2 μL, 25G⅝”: 7.5 μL ±1.3 μL, and 23G1¼”: 8.3 μL ±1.3 μL; p<0.0001; Fig 3A). In contrast, the 2 mL Nevershare^®^ syringes attached to the Total Dose™ LDS needles (25G⅝” LDS: 8.7 μL ±2.7 μL and 23G1¼” LDS: 9.6 μL ±3.1 μL) had residual volumes that were comparable to those of 1 mL tuberculin syringes attached to standard needles of identical sizes (25G⅝”: 7.5 μL ±1.3 μL and 23G1¼”: 8.3 μL ±1.3 μL; Fig 3A). The 2 mL Noloss LDS syringes attached to standard needles consistently retained significantly higher volumes of dye than 2 mL standard Nevershare^®^ syringes attached to Total Dose™ LDS needles of equivalent sizes (Noloss+25G⅝” standard > Nevershare^®^+25G⅝” LDS, p<0.0001; Noloss+23G1¼” standard > Nevershare^®^+23G1¼” LDS, p = 0.0002; Fig 3A).

### HCV infectivity in dead space volumes immediately after contamination

We then tested for residual HCV infectivity in the different syringe-needle pairs after loading with HCV-spiked plasma (Table 1). The 1 mL insulin syringes with fixed 27G½” needles had the lowest proportion of HCV-positive syringes (47%±4% HCV-positive syringes; Fig 3B). Among the syringes with detachable needles, the 2 mL Nevershare^®^ syringes with 23G1¼” LDS needles had the lowest percentage of HCV-positive syringes (65%±5% HCV-positive syringes) while those of the remaining syringes ranged from 80%±5% (2 mL Nevershare^®^ +25G⅝” LDS needle) to 98%±1% (2 mL Nevershare^®^+23G1¼” standard needle) HCV-positive syringes (Fig 3B).

**Table 1:** Comparison of HCV Recovery and Residual Infectivity Immediately after Contamination.

Syringe-Needle Combinations			HCV Recovery			Residual Infectivity	
High Dead Space	Low Dead Space	Insulin	# HCV+	% HCV+	p-value	RLU ± s.d.	p-value
Tuberculin +27G½"			52/60	87%	0.79	10030 ± 7790	0.16
	Chirana-Luer +27G½"		51/60	85%		7954 ± 7037	
Tuberculin +25G⅝"			53/60	88%	0.34	9339 ± 6841	0.02
	Chirana-Luer +25G⅝"		56/60	93%		12869 ± 9166	
Tuberculin +23G1¼"			57/60	95%	0.70	9861 ± 7026	0.009
	Chirana-Luer +23G1¼"		56/60	93%		14488 ± 10917	
Nevershare +25G⅝"			57/60	95%	0.01	17066 ± 8539	p<0.0001
	Nevershare +25G⅝"LDS		48/60	80%		5724 ± 4014	
Nevershare +23G1¼"			59/60	98%	p<0.0001	15245 ± 9346	p<0.0001
	Nevershare +23G1¼"LDS		39/60	65%		7127 ± 5406	
	Chirana-Luer +27G½"		51/60	85%	p<0.0001	7954 ± 7037	0.01
		Insulin 27G½”	42/90	47%		4978 ± 3570	
	Chirana-Luer +25G⅝"		56/60	93%	p<0.0001	12869 ± 9166	p<0.0001
		Insulin 27G½”	42/90	47%		4978 ± 3570	
	Chirana-Luer +23G1¼"		56/60	93%	p<0.0001	14488 ± 10917	p<0.0001
		Insulin 27G½”	42/90	47%		4978 ± 3570	
	Nevershare +25G⅝"LDS		48/60	80%	p<0.0001	5724 ± 4014	0.35
		Insulin 27G½”	42/90	47%		4978 ± 3570	
	Nevershare +23G1¼"LDS		39/60	65%	0.03	7127 ± 5406	0.04
		Insulin 27G½”	42/90	47%		4978 ± 3570	
Tuberculin +27G½”			52/60	87%	p<0.0001	10030 ± 7790	p<0.0001
		Insulin 27G½”	42/90	47%		4978 ± 3570	

The 1 mL insulin syringes with fixed 27G½” needles and the 2 mL Nevershare^®^ syringes attached to the 25G⅝” LDS needles had the lowest levels of residual infectivity (4978 RLU ±3570 RLU and 5724 RLU±4014 RLU respectively, Table 1). This was followed closely by that of the 2 mL Nevershare^®^ syringe-23G1¼” LDS needle combination (7127 RLU ±5406 RLU) and the 2 mL Noloss LDS syringe-27G½” standard needle combination (7954 RLU ±7037 RLU; Table 1). The remaining residual infectivity values ranged from 9339 RLU ±6841 RLU (1 mL tuberculin+25G⅝” standard needle) to 17066 RLU ±8539 RLU (2 mL Nevershare^®^+25G⅝” standard needle; Table 1).

### HCV infectivity in dead space volumes after storage

**Fig 4:**
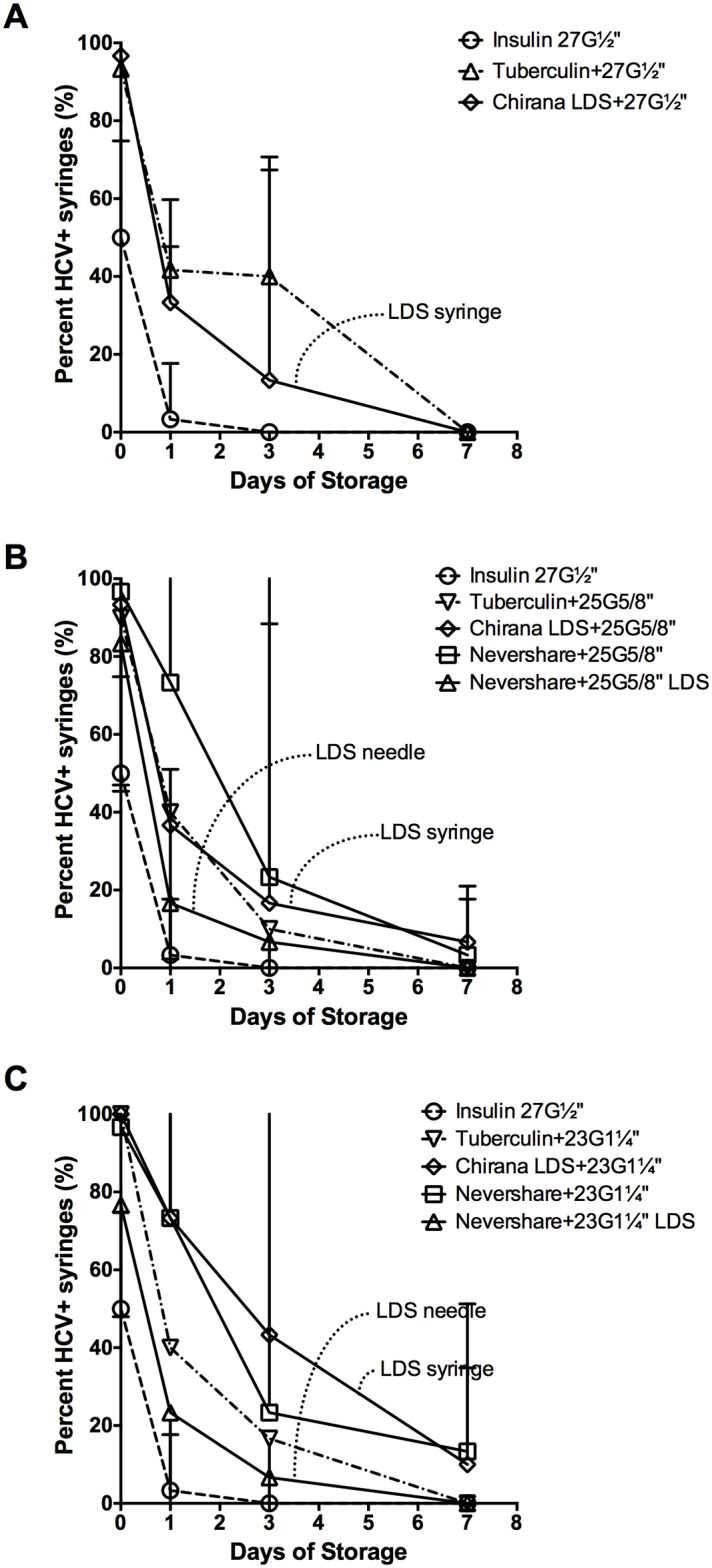
HCV stability in LDS and HDS syringe-needle combinations stored at room temperature. Syringe-needle pairs were loaded with plasma spiked with HCV and stored at room temperature for up to 1 week. The frequency of HCV-positive syringes with (A) 27-gauge, (B) 25-gauage, and (C) 23-gauge needle size was determined. Data from insulin syringes with fixed 27-gauge needles are repeated on each graph for comparison purposes (open circles and dashed lines). The percent HCV-positive syringes ±95% C.I. from at least 3 experiments are represented by each data point. G = gauge. LDS = low dead space.

HCV infectivity in all the different syringe-needle combinations declined during the course of storage at room temperature for up to one week (Fig 4). The 1 mL insulin syringes with fixed needles had the lowest number of HCV-positive syringes after the first day of storage (3%±2% HCV-positive syringes), with no detectable virus by day 3 (Fig 4A–4C). Despite similar residual infectivity at immediately after contamination (Table 1), 2 mL Noloss LDS syringes with 27G½” standard needles had significantly lower residual infectivity than the 1 mL tuberculin syringes attached to 27G½” standard needles at day 1 of storage at room temperature (Noloss: 1739 RLU±651 RLU, Tuberculin: 4194 RLU±2637 RLU, p<0.01; data not shown). By day 3 of storage, there were significantly fewer HCV-positive 2 mL Noloss LDS syringes with 27G½” standard needles than 1 mL tuberculin syringes with identical needles (Noloss: 13%±8%, Tuberculin: 40%±7% HCV-positive syringes, p<0.05; Fig 4A). When both the 2 mL Noloss and the 1 mL tuberculin syringes were attached to the larger bore 23G1¼” standard needles, significantly fewer HCV-positive 2 mL Noloss LDS syringes were recovered by day 1 of storage (Noloss: 73%±4%, Tuberculin: 40%±0% HCV-positive syringes, p<0.01; Fig 4C) and at day 3 of storage (Noloss: 43%±14%, Tuberculin: 17%±5% HCV-positive syringes, p<0.05; Fig 4C). The Noloss syringes also had significantly lower residual infectivity by day of storage (Noloss: 2184 RLU ±1829 RLU, Tuberculin: 4557 RLU±1724 RLU, p<0.05; data not shown).

Second only to the insulin syringes, syringes attached to the Total Dose™ LDS needles had the lowest number of HCV-positive syringes after 1 and 3 days of storage at room temperature (Figs 4B and 4C). 2 mL Nevershare^®^ standard syringes attached to 25G⅝” LDS needles had 2-fold fewer HCV-positive syringes than the 2 mL Noloss LDS syringes attached to 25G⅝” standard needles at day 1 of storage (Nevershare^®^+25G⅝” LDS: 17%±2% HCV-positive, Noloss LDS+25G⅝” standard: 37%±2% HCV-positive; Fig 3B). A similar pattern is observed with 23G1¼” needles, with a significant 3-fold difference at day 1 of storage (Nevershare^®^+23G1¼” LDS: 23%±4% HCV-positive, Noloss LDS+23G1¼” standard: 73%±4% HCV-positive, p<0.01; Fig 4C) and a significant 6-fold difference by day 3 of storage (Nevershare^®^+23G1¼” LDS: 7%±4% HCV-positive, Noloss LDS+23G1¼” standard: 43%±14% HCV-positive, p<0.01; Fig 4C).

Decay rates were calculated for the loss of infectivity with storage for all the different syringe-needle combinations (Table 2). Because of the limited number of replications, the 95% confidence intervals all overlap, so the decay rates cannot be construed to differ across conditions. However, it is worth noting that, as expected, the point estimate for the insulin syringes is the lowest and that LDS needles shortened half-lives compared to standard needles on the Nevershare syringes.

**Table 2:** Half-life and 95% Confidence Intervals for HCV Recovery Following Storage of HCV Contaminated Syringe/Needle Combinations.

	Syringe Type			
Needle Type	Insulin	Tuberculin	Noloss	Nevershare
27G, ½”	0.256	1.98	0.725	--
	(0.147–0.975)	(1.195–5.754)	(0.509–1.263)	
25G, **5/8”**	--	0.883	0.889	1.648
		(0.579–1.853)	(0.576–1.947)	(0.975–5.313)
25G, **5/8”** LDS	--	--	--	0.444
				(0.305–0.814)
23G, 1¼”	--	0.874	2.333	1.797
		(0.681–1.219)	(1.469–5.661)	(1.148–4.145)
23G, 1¼” LDS	--	--	--	0.612
				(0.429–1.069)

Notes:

Half-life is measured in days.

Abbreviations: G = gauge, LDS = low dead space

--Syringe/needle combinations not available for testing.

### Effect of needle size on HCV retention in different syringe-needle combinations

**Fig 5:**
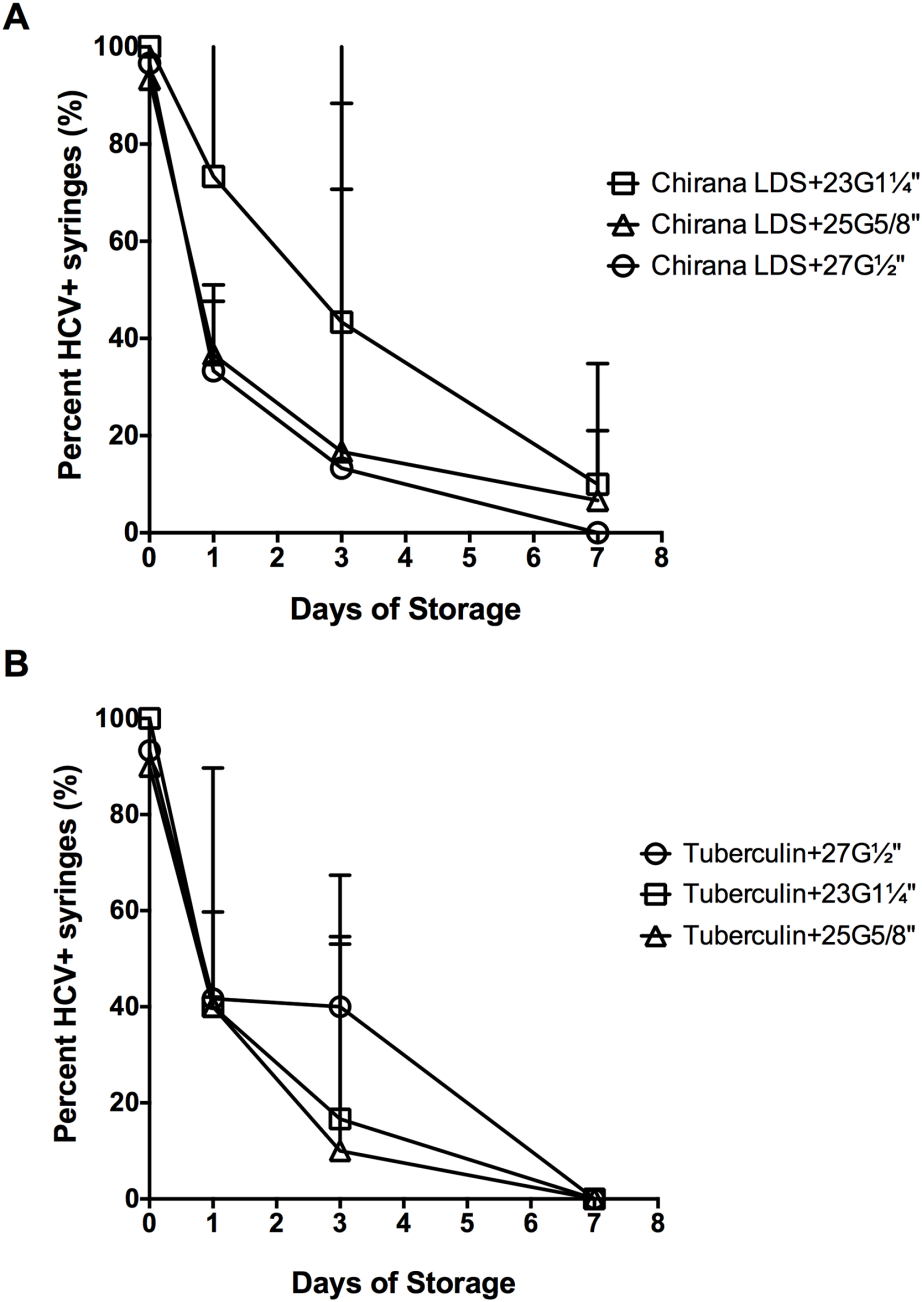
Effect of needle size on HCV retention in the syringes-needle combinations. Syringe-needle pairs were loaded with HCV-spiked plasma and stored at room temperature for up to 1 week. The percentage of HCV-positive (A) 2 mL Noloss LDS and (B) 1 mL tuberculin syringes was determined. Each data point denotes the percentage of HCV-positive syringes ±95% C.I. from at least 3 experiments. G = gauge. LDS = low dead space.

We compared HCV recovery across the three types of needles that differed in gauge and length (Fig 5). The 2 mL Noloss LDS syringes with 23G1¼” standard needles had the greatest proportion of HCV-positive syringes (Fig 5A) followed by the 25G⅝” and the 27G½” standard needles (Fig 5A). At day 1 of storage, HCV recovery was significantly lower in Noloss syringes attached to 27G½” (33%±2% HCV-positive) and the 25G⅝” (37%±2%) standard needles than in Noloss syringes with 23G1¼” (73%±4%) standard needles (Noloss+27G½” < Noloss+23G1¼”, p<0.05; Noloss+25G⅝” < Noloss+23G1¼”, p<0.01; Fig 5A). Residual infectivity was also significantly different according to needle size at day 1; with residual infectivity in Noloss syringes attached to 27G½” needles (1739 RLU±651 RLU) being significantly lower than when attached to 25G⅝” needles (3356 RLU±1895 RLU, p<0.05) and 23G1¼” needles (6225 RLU±5150 RLU, p<0.001; data not shown). This pattern was not observed for the 1 mL tuberculin syringes (Fig 5B).

### Effect of rinsing HCV-contaminated syringes with water

**Fig 6:**
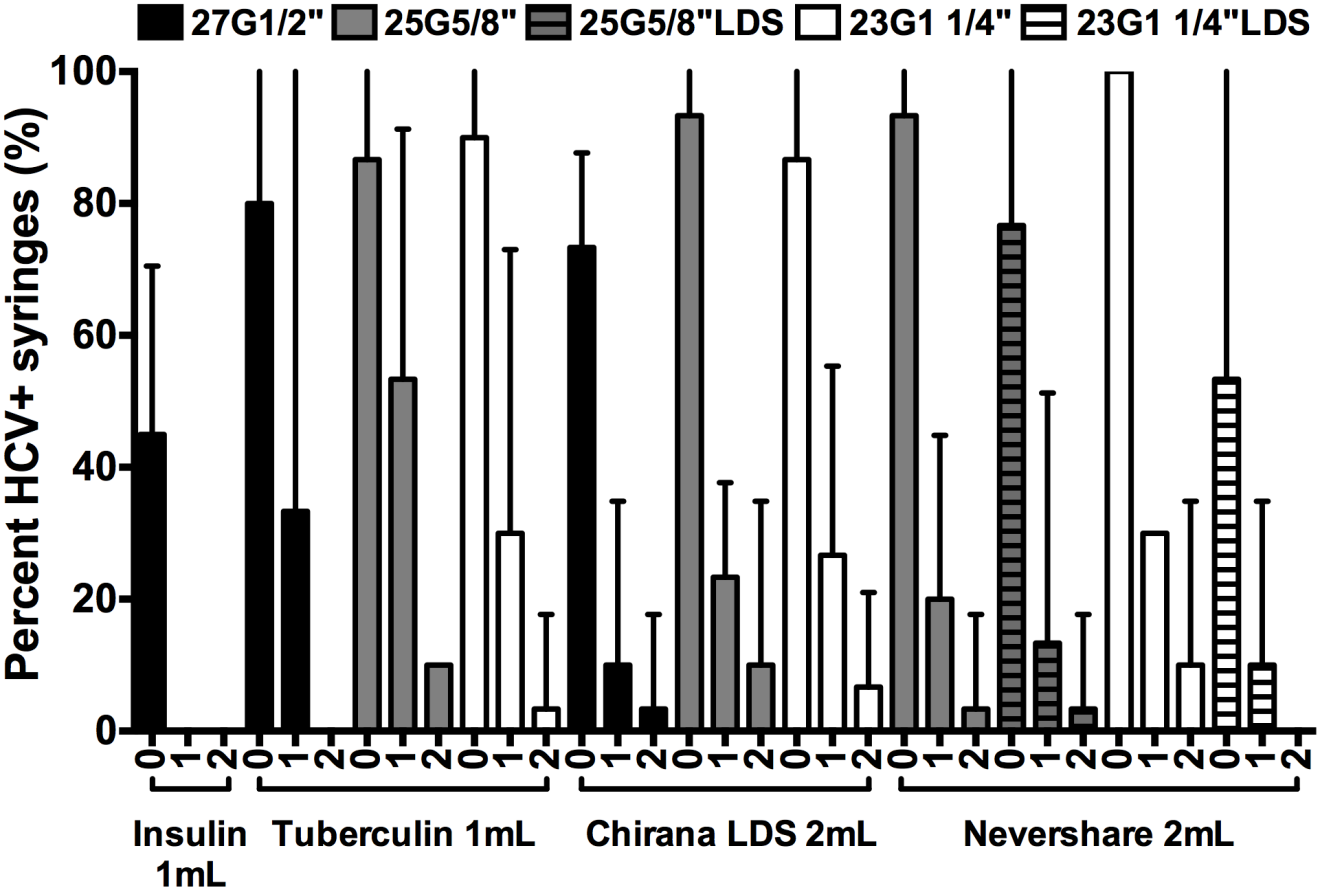
Residual viable HCV in LDS and HDS syringe-needle combinations after rinsing with water. Syringes were loaded with plasma spiked with HCV and rinsed once or twice with water and the frequency of HCV-positive syringes was determined. The percentage of HCV-positive syringes ±95% C.I. from at least 3 experiments are represented by each data point. G = Gauge. LDS = low dead space.

We determined HCV recovery after rinsing the syringe-needle combinations once or twice with water (Fig 6). In agreement with our previous report [24], one rinse with water reduced HCV infectivity to levels below the limit of detection in 1 mL insulin syringes with fixed 27G½” needles (Fig 6). The remaining syringe-needle combinations required multiple rinses to reduce residual infectivity to low levels (Fig 6). The 1 mL tuberculin syringes attached to PrecisionGlide™ 27G½” standard needles and the 2 mL Nevershare^®^ syringes with Total Dose™ LDS 23G1¼” needles were the only two syringe-needle combinations with detachable needles that yielded no HCV-positive syringes after two rinses with water (Fig 6).

## Discussion

Syringes with fixed needles, which retain the smallest volume after use, may not always be useful to or acceptable among PWID. Attempts to deal with this reality have led to the manufacture of detachable syringe-needle combinations that seek to reduce the dead space found in traditional detachable needle-syringe combinations. Our study confirms that the 1 mL insulin syringes with fixed 27G½” needles were the most effective of all injection equipment we tested at reducing HCV retention in syringes, outperforming all detachable syringe and needle combinations tested. This held true whether HCV recovery was assessed immediately after use, after storage at room temperature, or after rinsing with water. Our findings suggest that despite the changes in syringe and needle design to offer low dead space options, more improvements are needed if HCV transmission is to be reduced. We have demonstrated that the Noloss LDS syringes with standard needles and the standard Nevershare^®^ syringes with Total Dose™ LDS needles retained levels of viable HCV that were comparable to their HDS counterparts immediately after use.

Upon storage at room temperature, however, distinctions between the detachable LDS syringe-needle pairs were observed. The Nevershare^®^ standard syringes attached to the Total Dose™ LDS needles showed a faster decline over time in HCV recovery at room temperature than the Noloss LDS syringes, consistently yielding the lowest proportion of HCV-positive among the syringes with detachable needles during the week of storage at room temperature. These results suggest possible differences in the impact of the LDS syringes and needles on reducing infectious HCV in syringes, but only upon substantial lag times between uses.

Overall, however, the lack of a consistent pattern across all three sets of experiments—immediate testing, storage, and rinsing with water—and the variance between experiments preclude the recommendation of any one LDS syringe-needle combination over the others or in place od HDS syringes. Given that the differences in cost between standard and the lower dead space syringe/needle combinations are minimal and that the lower dead space syringes cost more without providing much benefit suggest little or no economic benefit in providing the lower dead space syringes as an HCV prevention measure. Instead, HCV prevention efforts should emphasize the importance of traditional harm reduction approaches that include preparing drugs and injecting them only with new, sterile equipment, minimizing contamination of injection locales, and hypochlorite bleach disinfection [21, 24–26].

Our study has several limitations. First, our experiments were done solely at room temperature, which potentially restricts the relevance of our findings to PWID who are able to store their used syringes at approximately 22°C. Based on our previously published work, we predict that storage at lower temperatures would slow the rate of loss of HCV viability and higher temperatures would increase the rate [20, 21, 24,27]. We hope to address the effect of temperature in future studies. Second, our use of plasma instead of blood could affect our findings as the thicker consistency of blood could influence virus retention or survival in the dead space of the syringes. Third, the use of a genotype 2a laboratory clone of HCV may have survival characteristics different from other genotypes. One this last point, a previous study with HCV clones of different genotypes has shown HCV thermostability to be relatively similar across genotypes [28], suggesting that our findings could be generalized. Fourth, we tested limited quantities and specific brands of LDS injection equipment, so we are unable to determine if other designs out-perform those we tested.

The duration of HCV recovery from tuberculin syringes appeared shorter than in studies we previously published [20]. We have no firm explanation for the more rapid decline. Possible explanations include batch-to-batch variations in HCV titer, differences in permissibility of the Huh-7.5 subclones grown out from storage, or variations in the human plasma used to dilute HCV stocks and load syringes and growth factors contained in the fetal bovine serum used to supplement culture medium. It should be noted that the earlier set of experiments, which involved far more syringes per condition, revealed a biphasic decay pattern in which the second phase was far less steep, accounting for the prolonged infectivity in a small percentage of syringes. The decay rates we report herein for tuberculin syringes are consistent with the decay rates for the first phase we reported in 2010.

There remains much that is unknown about the overall impact of syringe type on HCV transmission among PWID. Our findings suggest that the low volume fixed needle 1 mL insulin syringe continues to be the best option for limiting HCV transmission. PWID who require the of use syringes with larger volumes and detachable needles need to be made aware that they are at increased risk for HCV transmission compared to fixed needle syringes if injection equipment is reused, even when using two-part LDS syringes and even after several rinses with water. Additional efforts are needed to improve the design of LDS needles and syringes to reduce the risk of HCV transmission and further laboratory research is necessary to determine whether LDS syringe use could be beneficial in reducing the transmission of other blood borne pathogens, especially HIV, among PWID.

## Supporting Information

S1 File Data for Figs 3–6. (XLSX)

## References

[1] MacArthur GJ, van Velzen E, Palmateer N, Kimber J, Pharris A, Hope V, et al Interventions to prevent HIV and Hepatitis C in people who inject drugs: A review of reviews to assess evidence of effectiveness. International Journal of Drug Policy. 2014;25(1):34–52. 10.1016/j.drugpo.2013.07.001 23973009

[2] Hagan LM, Schinazi RF. Best strategies for global HCV eradication. Liver International. 2013;33:68–79. 10.1111/liv.12063 23286849 PMC4110680

[3] Shepard CW, Finelli L, Alter MJ. Global epidemiology of hepatitis C virus infection. The Lancet Infectious Diseases. 2005;5(9):558–67. 10.1016/S1473-3099(05)70216-4 16122679

[4] Murray CL, Rice CM. Turning Hepatitis C into a Real Virus. Annual Review of Microbiology. 2011;65(1):307–27. 10.1146/annurev-micro-090110-102954 21682640

[5] De P, Roy É, Boivin JF, Cox J, Morissette C. Risk of hepatitis C virus transmission through drug preparation equipment: a systematic and methodological review. Journal of Viral Hepatitis. 2008;15(4):279–92. 10.1111/j.1365-2893.2007.00942.x 18208496 PMC2929252

[6] Paintsil E, Verevochkin SV, Dukhovlinova E, Niccolai L, Barbour R, White E, et al Hepatitis C virus infection among drug injectors in St Petersburg, Russia: social and molecular epidemiology of an endemic infection. Addiction. 2009;104(11):1881–90. Epub 2009/08/29. 10.1111/j.1360-0443.2009.02687.x 19712125 PMC2763027

[7] Smith BD, Jewett A, Burt RD, Zibbell JE, Yartel AK, DiNenno E. “To Share or Not to Share?” Serosorting by Hepatitis C Status in the Sharing of Drug Injection Equipment Among NHBS-IDU2 Participants. Journal of Infectious Diseases. 2013;208(12):1934–42. 10.1093/infdis/jit520 24136794 PMC5759767

[8] Palmateer N, Hutchinson S, McAllister G, Munro A, Cameron S, Goldberg D, et al Risk of transmission associated with sharing drug injecting paraphernalia: analysis of recent hepatitis C virus (HCV) infection using cross-sectional survey data. Journal of Viral Hepatitis. 2014;21(1):25–32. 10.1111/jvh.12117 24329854

[9] Zule WA, Desmond DP. Various Types of Injection Equipment and Risk of HIV Infection. JAIDS Journal of Acquired Immune Deficiency Syndromes. 1997;16(4):309. 10.1097/00042560-199712010-00015 9402081

[10] Zule WA, Bobashev G. High dead-space syringes and the risk of HIV and HCV infection among injecting drug users. Drug and Alcohol Dependence. 2009;100(3):204–13. 10.1016/j.drugalcdep.2008.08.017 19004579 PMC2654245

[11] Zule WA, Cross HE, Stover J, Pretorius C. Are major reductions in new HIV infections possible with people who inject drugs? The case for low dead-space syringes in highly affected countries. International Journal of Drug Policy. 2013;24(1):1–7. 10.1016/j.drugpo.2012.07.002 22884539

[12] Ibragimov U, Latypov A. Needle and syringe types used by people who inject drugs in Eastern Europe and Central Asia: Key findings from a rapid situational assessment. Eurasian Harm Reduction Network. 2012.

[13] Gray R, Tuan NM, Neukom J. Rapid assessment of needle and syringe types used by people who inject drugs in Hanoi and Ho Chi Minh City. Population Services International, Vietnam. 2012.

[14] Zule WA, Ticknor-Stellato KM, Desmond DP, Vogtsberger KN. Evaluation of Needle and Syringe Combinations. JAIDS Journal of Acquired Immune Deficiency Syndromes. 1997;14(3):294–7. 10.1097/00042560-199703010-00015 9117464

[15] Exchange Supplies. http://www.exchangesupplies.org Last accessed March 6, 2015.

[16] Apothicom. http://www.apothicom.org Last accessed March 6, 2015.

[17] Lindenbach BD, Meuleman P, Ploss A, Vanwolleghem T, Syder AJ, McKeating JA, et al Cell culture-grown hepatitis C virus is infectious in vivo and can be recultured in vitro. Proc Natl Acad Sci U S A. 2006;103(10):3805–9. . 16484368 10.1073/pnas.0511218103 PMC1533780

[18] Lindenbach BD, Evans MJ, Syder AJ, Wolk B, Tellinghuisen TL, Liu CC, et al Complete replication of hepatitis C virus in cell culture. Science. 2005;309(5734):623–6. Epub 2005/06/11. 10.1126/science.1114016 . 15947137

[19] Phan T, Beran RKF, Peters C, Lorenz IC, Lindenbach BD. Hepatitis C Virus NS2 Protein Contributes to Virus Particle Assembly via Opposing Epistatic Interactions with the E1-E2 Glycoprotein and NS3-NS4A Enzyme Complexes. Journal of Virology. 2009;83(17):8379–95. 10.1128/jvi.00891-09 19515772 PMC2738163

[20] Paintsil E, He H, Peters C, Lindenbach BD, Heimer R. Survival of Hepatitis C Virus in Syringes: Implication for Transmission among Injection Drug Users. Journal of Infectious Diseases. 2010;202(7):984–90. 10.1086/656212 20726768 PMC2932767

[21] Paintsil E, Binka M, Patel A, Lindenbach BD, Heimer R. Hepatitis C Virus Maintains Infectivity for Weeks After Drying on Inanimate Surfaces at Room Temperature: Implications for Risks of Transmission. Journal of Infectious Diseases. 2014;209(8):1205–11. 10.1093/infdis/jit648 24273176 PMC3969546

[22] Blight KJ, McKeating JA, Rice CM. Highly permissive cell lines for subgenomic and genomic hepatitis C virus RNA replication. J Virol. 2002;76(24):13001–14. . 12438626 10.1128/JVI.76.24.13001-13014.2002 PMC136668

[23] Sigma-Aldrich. Syringe Needle Gauge Chart. http://www.sigmaaldrich.com/chemistry/stockroom-reagents/learning-center/technical-library/needle-gauge-chart.html. Last accessed March 6, 2015.

[24] Binka M, Paintsil E, Patel A, Lindenbach BD, Heimer R. Disinfection of Syringes Contaminated With Hepatitis C Virus by Rinsing With Household Products. Open Forum Infectious Diseases. 2015;2(1). 10.1093/ofid/ofv017 PMC4438897 26034767

[25] Ellendon N. Hepatitis C Counseling Best Practices Manual. New York, NY: Harm Reduction Coaltion. Available from: http://harmreduction.org/hepatitis-c/hepatitis-tools/hepatitis-c-counseling-best-practices-manual/.

[26] Anonymous. Hep C: A handbook for Dorchester, UK: Exchange Supplies; 2009. Available from: http://www.exchangesupplies.org/shopdisp_HRPUB1.php?page=read.

[27] Ciesek S, Friesland M, Steinmann J, Becker B, Wedemeyer H, Manns MP et al How stable Is the hepatitis C virus (HCV)? Environmental stability of HCV and its susceptibility to chemical biocides. Journal of Infectious Diseases. 2010;201 (12): 1859–1866. 10.1086/652803 20441517

[28] Doerrbecker J, Meuleman P, Kang J, Riebesehl N, Wilhelm C, Friesland M, et al Thermostability of seven hepatitis C virus genotypes in vitro and in vivo. Journal of Viral Hepatitis. 2013;20(7):478–85. 10.1111/jvh.12055 23730841

