# Correction: Survival of Hepatitis C Virus in Syringes Is Dependent on the Design of the Syringe-Needle and Dead Space Volume

**DOI:** 10.1371/journal.pone.0146088

**Published:** 2015-12-23

**Authors:** Mawuena Binka, Elijah Paintsil, Amisha Patel, Brett D. Lindenbach, Robert Heimer

Fig 2 is incorrect. It is inadvertently a duplicate of Fig 3. Please view the corrected Fig 2 here.

**Fig 2:**
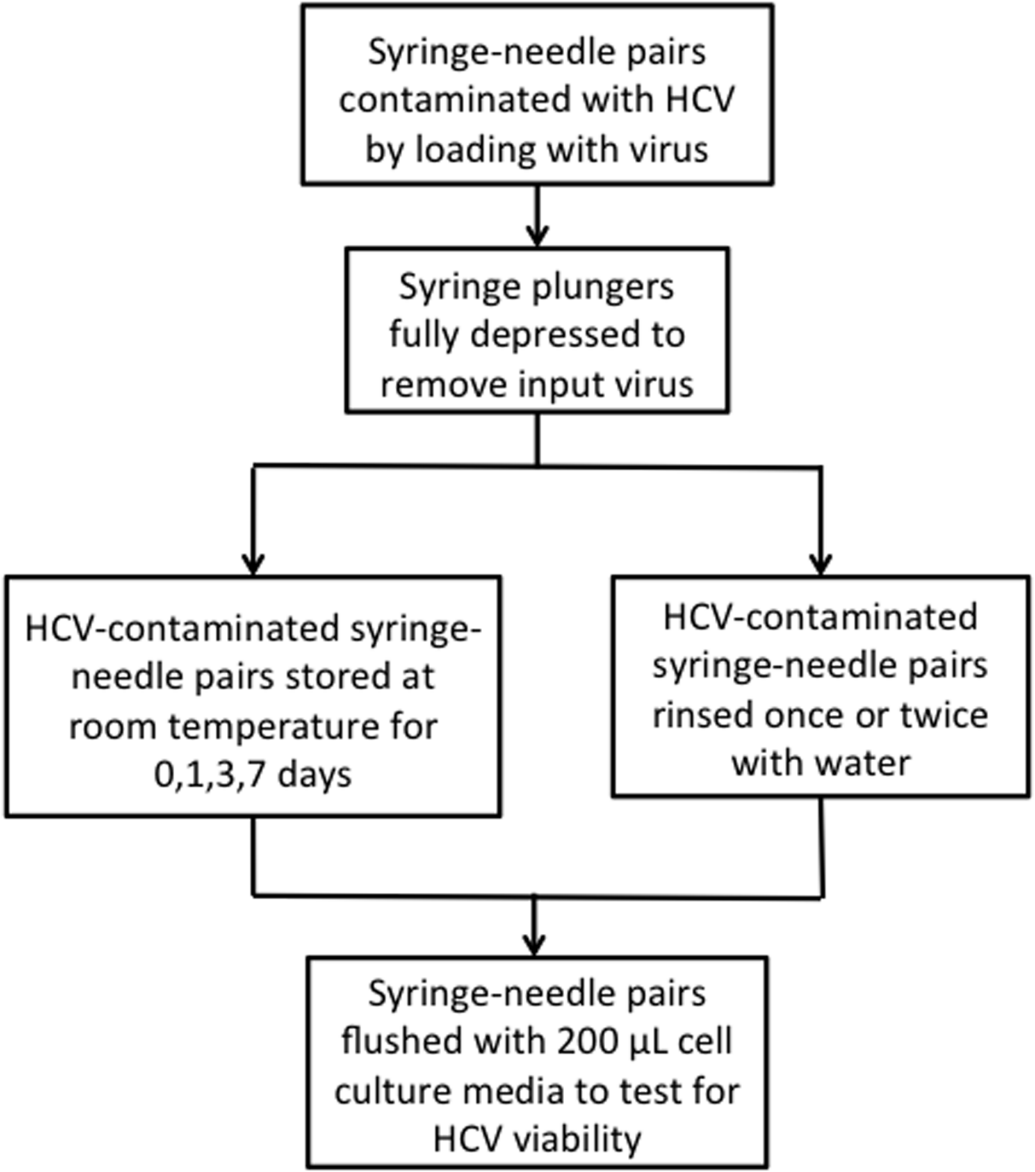
Flow diagram of syringe testing. Syringe-needle pairs were contaminated with virus and tested for viable HCV immediately after contamination, after storage at room temperature or after rinsing with water.
